# Biocompatible Poly(acrylic acid-*co*-methacrylic acid)-Coated Iron Oxide Nanoparticles for Enhanced Adsorption and Antimicrobial Activity of Lasioglossin-III

**DOI:** 10.1021/acsami.4c22603

**Published:** 2025-03-05

**Authors:** Marco Reindl, Verena Zach, Sebastian P. Schwaminger

**Affiliations:** † NanoLab, Division of Medicinal Chemistry, Otto Loewi Research Center, Medical University of Graz, Neue Stiftingtalstraße 6, 8020 Graz, Austria; § BioTechMed-Graz, Mozartgasse 12/II, 8010 Graz, Austria

**Keywords:** antibacterial activity, drug delivery, iron oxide nanoparticles, lasioglossin-III, polymer coating

## Abstract

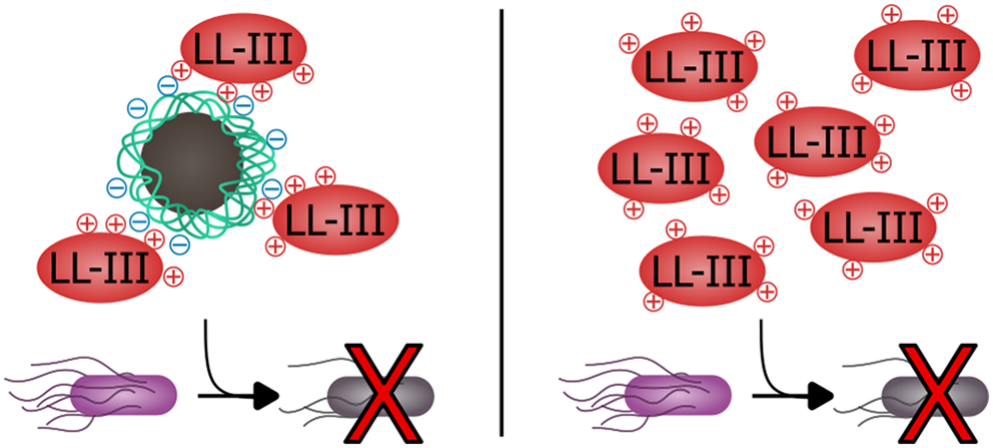

The development of biocompatible and efficient drug delivery platforms is critical for therapeutic applications. This study investigates poly(acrylic acid-*co*-methacrylic acid)-coated iron oxide nanoparticles [ION@P(AA-*co*-MAA)] as a delivery system for the cationic antimicrobial peptide lasioglossin-III (LL-III). Iron oxide nanoparticles (IONPs) were synthesized via coprecipitation and analyzed by transmission electron microscopy, dynamic light scattering (DLS), and vibrating sample magnetometry. The coating of IONPs was performed in situ, ensuring strong polymer adhesion to the iron oxide core and functionalization with carboxy groups for peptide adsorption. The hydrodynamic diameter of polymer-coated IONPs was determined by DLS and the polymer coating was confirmed by attenuated total reflectance-Fourier transform infrared (ATR-FTIR) spectroscopy through functional group signatures. ζ-Potential measurements revealed a strongly negative surface charge under physiological pH suggesting excellent colloidal stability. Investigation of LL-III adsorption on ION@P(AA-*co*-MAA) demonstrated a fast and efficient loading with 0.82 g/g at the highest investigated concentration (4 g/L LL-II), highlighting a superior adsorption efficiency compared to existing IONPs systems. After three washing steps with PBS, 49% of the peptide remained bound to the nanoparticles, indicating a stable adsorption of LL-III on the particles, markedly outperforming other IONP-based systems. The customizable polymer coating design enabled optimal peptide interactions, ensuring efficient loading and retention. Cytotoxicity studies suggested that both unloaded, and LL-III-loaded nanoparticles are biocompatible with 3T3 and HEK cells. Antimicrobial assays revealed enhanced LL-III efficacy upon nanoparticle adsorption, reducing the minimum inhibitory concentration (MIC) against *Escherichia coli* from 9.82 μM (free LL-III) to 4.59 μM for LL-III-loaded nanoparticles. These findings highlight ION@P(AA-*co*-MAA) as a promising drug delivery platform offering biocompatibility and enhanced antimicrobial efficacy laying a solid foundation for the development of advanced nanoparticle-based targeted therapies.

## Introduction

Nanotechnology has revolutionized medicine, where nanoparticles are increasingly recognized for their unique properties and versatility in applications such as imaging, diagnostics, and drug delivery.^1,2^ Among these, iron oxide nanoparticles (IONPs) have gained significant attention due to their superparamagnetic properties, biocompatibility, and low production costs.^3^ Their small size, nonporosity, ease of modification, and high surface area make them ideal for applications in diagnostics and imaging as well as carriers for therapeutic agents facilitating targeted delivery while minimizing side effects.^4−6^

IONPs are increasingly being used in a wide range of medical fields, from diagnostics and imaging to therapeutic applications. One example is magnetic particle imaging (MPI), a novel technique that uses superparamagnetic nanoparticles as tracers. MPI offers advantages such as linear quantitation, positive contrast, no radiation, unlimited penetration, and no background interference, making it ideal for cell tracking, tumor imaging, blood pool imaging, and magnetic hyperthermia. Although MPI is still in its early stages, advances have been made in long-term cell tracking and multimodal imaging.^5^ Another promising technology is magnetic beads, which carry biological recognition molecules to capture specific biomarkers and can also act as signal outputs in detection. Magnetic beads are inexpensive, provide rapid detection, and offer simple procedures compared to traditional immune-based methods. A common detection approach, magnetic relaxation switching, involves the aggregation or disaggregation of beads induced by the target. Magnetic beads have a wide range of applications, including cell sorting, nucleic acid separation, and immunoassays such as electrochemical, optical, and direct magnetic immunoassays.^6^ Both MPI and magnetic beads are only two examples of innovative diagnostic and therapeutic approaches exploiting IONPs in medicine.

IONPs are often synthesized using the Massart process, which is cost-effective and easy to perform, producing IONPs with high magnetization and sizes ranging from 4 to 16 nm making IONPs an attractive option for biomedical applications.^3,7^ These particles can be further coated with various materials, such as silica,^8^ lipids,^9^ or polymers,^10^ creating core–shell structures that enhance stability and allow for the tailoring of properties for use in biomedical applications.^11^ For targeted drug delivery, a drug can either be attached to the coated or functionalized particles, or it can be embedded within the coating matrix.^4,12^

Polymeric coatings, in particular, can stabilize the particles, prevent premature immunogenicity, and can facilitate the generation of a controlled release mechanism.^1,3^ They offer an improved dispersibility in biological fluids and enhanced biocompatibility, which are critical for therapeutic applications.^13^ Polymer coatings also act as a barrier to protect the iron oxide core from degradation or interaction with biological components and its environment.^14^ Additionally, they can reduce the immunogenicity of various drugs by shielding them from the immune system, thereby minimizing adverse immune responses.^15^ Importantly, the choice of polymer and its properties can be tailored to suit specific applications, allowing for the customization of drug delivery systems making polymer-coated IONPs a promising platform for advanced drug delivery systems.^15^

Acrylic acid (AA) and methacrylic acid (MAA) are structurally similar anionic monomers. Both monomers contain a carboxylic acid group that imparts a negative charge when ionized. This negative charge enables strong electrostatic interactions with cationic ligands, facilitating drug binding. However, MAA has an additional methyl group on the α-carbon increasing its steric hindrance and hydrophobic character compared to AA.^16^ Due to these characteristics, copolymers of AA and MAA exhibit a more uniform distribution of carboxylate groups, improving the accessibility of these functional sites for ion binding facilitating easier interaction between ions and functional groups, thereby improving the ion-exchange capacity.^17,18^

AA- and MAA-based polymers are EMA and FDA approved^19−21^ which supports the development of stable, functional coatings that improve drug loading efficiency and ensure safe, effective delivery. Poly(acrylic acid) (PAA), mostly used in its cross-linked form also known as Carbomer, is used in various drug delivery systems, including eye drop formulations with cyclosporine for dry eye disease^22^ and as hydrogels as a carrier for corticosteroids as well as mucoadhesive nasal gels to extend the contact of corticosteroids with nasal mucosa, enabling controlled drug release.^23^ Poly(methacrylic acid) (PMAA) is commonly used in drug delivery as a copolymer, also known under its trade name Eudragit.^24^ It has been employed for coating of encapsulated drugs and to deliver a variety of drugs, including anti-inflammatory agents (e.g., diclofenac and ibuprofen), the antifungal amphotericin B, and the antibiotic vancomycin.^25^

To optimize polymer-coated nanoparticles for drug delivery, understanding adsorption and desorption mechanisms is essential, especially electrostatic interactions that can control the adsorption of charged biomolecules.^26^ This is particularly relevant for antimicrobial peptides, such as lasioglossins, known for their antimicrobial properties making them promising candidates for alternatives to traditional antibiotics.^27^ Lasioglossin-III (LL-III) is a cationic, α-helical peptide that interacts with negatively charged microbial membranes, disrupting them and causing cell death.^27,28^ Recent studies investigated the adsorption capacity and kinetics of LL-III on bare and silica-coated IONPs for controlled drug delivery.^7,8^ While LL-III showed promise as a model peptide for such studies, the adsorption capacity and desorption resistance on bare and silica-coated IONPs was insufficient.^7,8^

In this study, we examined the adsorption and desorption of LL-III on IONPs coated with PAA, PMAA, and poly(acrylic acid-*co*-methacrylic acid) [P(AA-*co*-MAA)], hypothesizing that negatively charged coatings would improve peptide adsorption. We tested three monomer formulations, evaluated adsorption dynamics, and used transmission electron microscopy (TEM), dynamic light scattering (DLS), and attenuated total reflectance-Fourier transform infrared (ATR-FTIR) to analyze nanoparticle properties. The ION@P(AA-*co*-MAA) formulation showed the best peptide adsorption, which was further tested for biocompatibility and antibacterial effects, aiming to optimize nanoparticle systems for drug delivery.

## Results and Discussion

### Synthesis and Physicochemical Characterization of Bare and Polymer-Coated Iron Oxide Nanoparticles

IONPs were prepared according to a previously published protocol by Turrina et al.^7^ and serve as a platform for the subsequent coating with anionic polymers. The IONPs were characterized by key attributes such as particle size, surface composition, crystalline phase, and magnetization properties.

**Figure 1:**
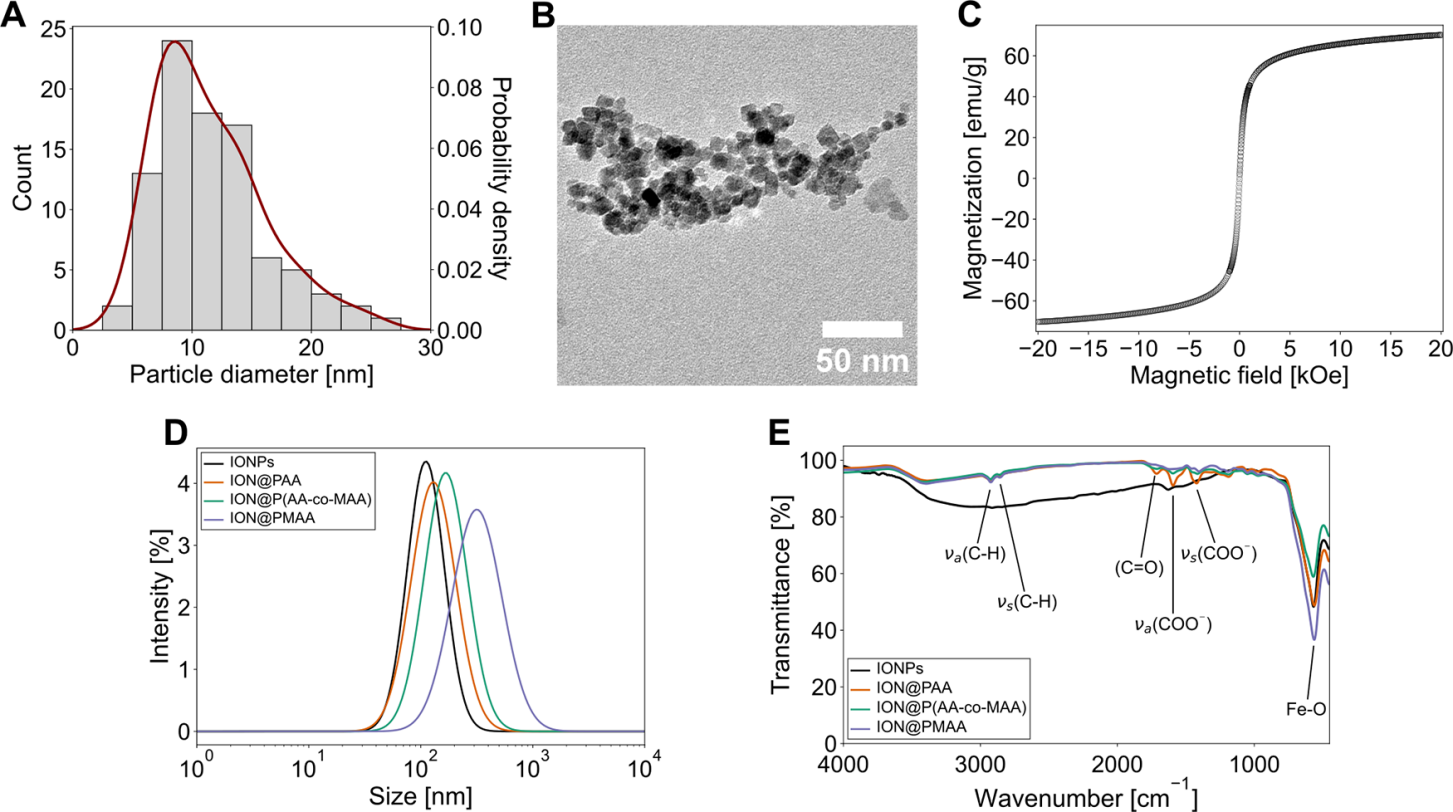
Physicochemical characterization of bare iron oxide nanoparticles (IONPs), PAA-coated IONPs (ION@PAA), P(AA-*co*-MAA)-coated IONPs [ION@P(AA-*co*-MAA)], and PMAA-coated IONPs (ION@PMAA). (A) Distribution of particle diameters as determined by transmission electron microscopy (TEM) with the respective probability density (red), *n* = 90, *d* = 11.5 ± 4.5 nm (PDI 1.15). (B) Representative TEM micrograph of IONPs. (C) Magnetization curve of IONPs determined by vibrating sample magnetometry at 298 K. (D) Hydrodynamic diameter determined by dynamic light scattering (DLS) in ultrapure water (pH 7.2) at room temperature with intensity-weighed size distribution. (E) Chemical composition analysis performed using attenuated total reflectance Fourier-transform infrared spectroscopy (ATR-FTIR) obtained at room temperature, highlighting characteristic functional groups associated with the polymer coatings and IONPs.

The particle size of IONPs was determined by TEM, which revealed an average diameter of 11.5 ± 4.5 nm and a PDI of 1.15, indicating a moderate uniformity (Figure 1A,B). Size and PDI are similar to previously obtained measurements of IONPs synthesized by coprecipitation which reported particle size distributions between 6 and 14 nm.^7,29^ The hydrodynamic diameter (Z-average diameter) of the nanoparticles was determined by DLS to be 89 nm with a PDI of 0.156 (Figure 1D and Table 1). The observed discrepancy between the particle size measurements obtained via TEM and DLS can be attributed to several factors. TEM measures the dry diameter of nanoparticles, while DLS evaluates the hydrodynamic diameter in solution, which is influenced by interactions with the surrounding medium. Additionally, factors such as variations in contrast and potential particle aggregation can further contribute to this difference. It is also important to note that TEM typically reports a smaller size due to the drying process and inherent resolution limitations during imaging.^30^

**Table 1:** Summary of the Z-Average Diameter and PDI of the Particles as Determined by Dynamic Light Scattering (DLS)^a^

particles	Z-average diameter [nm]	PDI
IONPs	89	0.156
ION@PAA	130	0.234
ION@P(AA-*co*-MAA)	137	0.216
ION@PMAA	212	0.304

a Measurements were performed in ultrapure water at room temperature with a particle concentration of 50 mg/L.

The superparamagnetic properties of IONPs were evaluated using vibrating sample magnetometry (VSM). The synthesized particles demonstrated the characteristic sigmoidal magnetization curve of superparamagnetic nanoparticles (Figure 1C), showing no hysteresis or remanent magnetization at 0 Oe.^31^ The maximum magnetization was reached at 69.9 emu/g which aligns with the magnetization values obtained in earlier studies.^7,29^ Even though the magnetization of the particles shows the typical behavior for superparamagnetic materials, the presence of slopes at high magnetic fields suggests the existence of paramagnetic material within the particles which can be explained by the presence of free paramagnetic iron ions, water residues, or oxygen in the sample.^32^

X-ray photoelectron spectroscopy (XPS) was used to characterize the surface composition and oxidation states of the iron ions (Figure S1A). The two closely spaced peaks at about 710 eV can be assigned to Fe 2p_1/2_ and Fe 2p_3/2_ indicating the presence of bivalent (Fe^2+^) and trivalent (Fe^3+^) iron ions, which is a common feature in mixed valence state IONPs, such as magnetite (Fe_3_O_4_). The peak around 530 eV can be attributed to the O 1s core which represents the oxygen atoms in the material. This is a characteristic peak for metal oxides such as Fe_2_O_3_ and Fe_3_O_4_.^33^ The crystalline phase of the particles was identified by X-ray diffraction (XRD), which showed the characteristic reflections for cubic magnetite at 220, 311, 400, 511, and 440 (Figure S1B).^7^

The synthesized IONPs exhibited key properties such as moderate particle size distribution (Figure 1A,B,D), superparamagnetic behavior (Figure 1C), and crystalline structure consistent with magnetite (Figure S1B). XPS and XRD analyses confirmed the presence of mixed valence iron states (Fe^2+^ and Fe^3+^) and the expected cubic magnetite phase (Figure S1A,B).

The polymer coating of the previously synthesized IONPs was carried out using modified versions of two established protocols.^10,13^ In this process, the IONPs were combined with SDS, which acts as surfactant, and with the appropriate monomers, i.e., AA, MAA, or a combination of both. This coating procedure is straightforward and cost-effective, providing an efficient approach to modify the surface properties of the IONPs and enhancing their suitability for the delivery of positively charged drugs. Three different polymer coatings – PAA, PMAA, and P(AA-*co*-MAA) – were applied to investigate their impact on particle size, drug adsorption, and loading stability.

The hydrodynamic diameter (Z-average diameter) was determined to be 130 nm (PDI 0.234) for PAA-coated IONPs (ION@PAA), 212 nm (PDI 0.304) for PMAA-coated IONPs (ION@PMAA), and 137 nm (PDI 0.216) for P(AA-*co*-MAA)-coated IONPs [ION@P(AA-*co*-MAA)] (Figure 1D, Table 1). These measurements align with previously reported hydrodynamic diameters for polymer-coated IONPs.^13^ The data also provide valuable insights into the impact of different coatings. ION@PAA exhibited a relatively narrow size distribution, while ION@PMAA displayed a higher PDI, indicating a broader size range. In contrast, ION@P(AA-*co*-MAA) showed a hydrodynamic diameter similar to that of the PAA-coated particles, with minor variations due to the combination of both monomers. These differences in size and PDI may influence the colloidal stability, biodistribution, and uptake efficiency of the nanoparticles in biological systems.^34,35^

The chemical composition of the polymer-coated IONPs was analyzed by attenuated total reflectance Fourier-transform infrared spectroscopy (ATR-FTIR) which revealed several key absorption peaks corresponding to specific molecular vibrations (Figure 1E and Table 2). The prominent band at 565 cm^–1^ can be attributed to a vibrational mode of Fe–O, characteristic of iron oxide nanoparticles which are spinel based.^36^ Characteristic peaks for carboxylic acid at 1438 cm^–1^ corresponding to ν_s_(COO^–^), and at 1593 cm^–1^ corresponding to ν_a_(COO^–^) indicate the presence of the group in its deprotonated form.^10^ At 1725 cm^–1^, ν(C=O) of the protonated carboxylic acid group can be observed.^37^ Additionally, peaks at 2856 and 2928 cm^–1^, corresponding to ν_s_(C–H) and ν_a_(C–H), typical of methyl and methylene groups, are present, indicating the presence of PAA, PMAA, or both.^10,38^

**Table 2:** Summary of the Assignment of Peaks in the ATR-FTIR Spectra of Figure 1E

wavenumber [cm^–1^]	assignment
565	(Fe–O)^36^
1438	ν_s_(COO^–^)^10^
1593	ν_a_(COO^–^)^10^
1725	ν(C=O)
2856	ν_s_(C–H)^10,38^
2928	ν_a_(C–H)^10,38^

The physicochemical characterization of the polymer-coated nanoparticles confirms the successful synthesis and coating process. The different particle sizes, size distributions (Figure 1D and Table 1), and chemical compositions (Figure 1E and Table 2) suggest that each polymer coating results in distinct, though related, properties for the nanoparticles. Additionally, ATR-FTIR spectra revealed the presence of key functional groups from the coatings, providing insights into polymer integration on the nanoparticle surfaces (Figure 1E and Table 2). These findings suggest that the polymerization strategy effectively functionalizes the surface of the nanoparticles, enhancing their suitability for applications in the delivery of cationic drugs.

### Adsorption and Desorption of LL-III on Differently Coated and Noncoated Iron Oxide Nanoparticles

**Figure 2:**
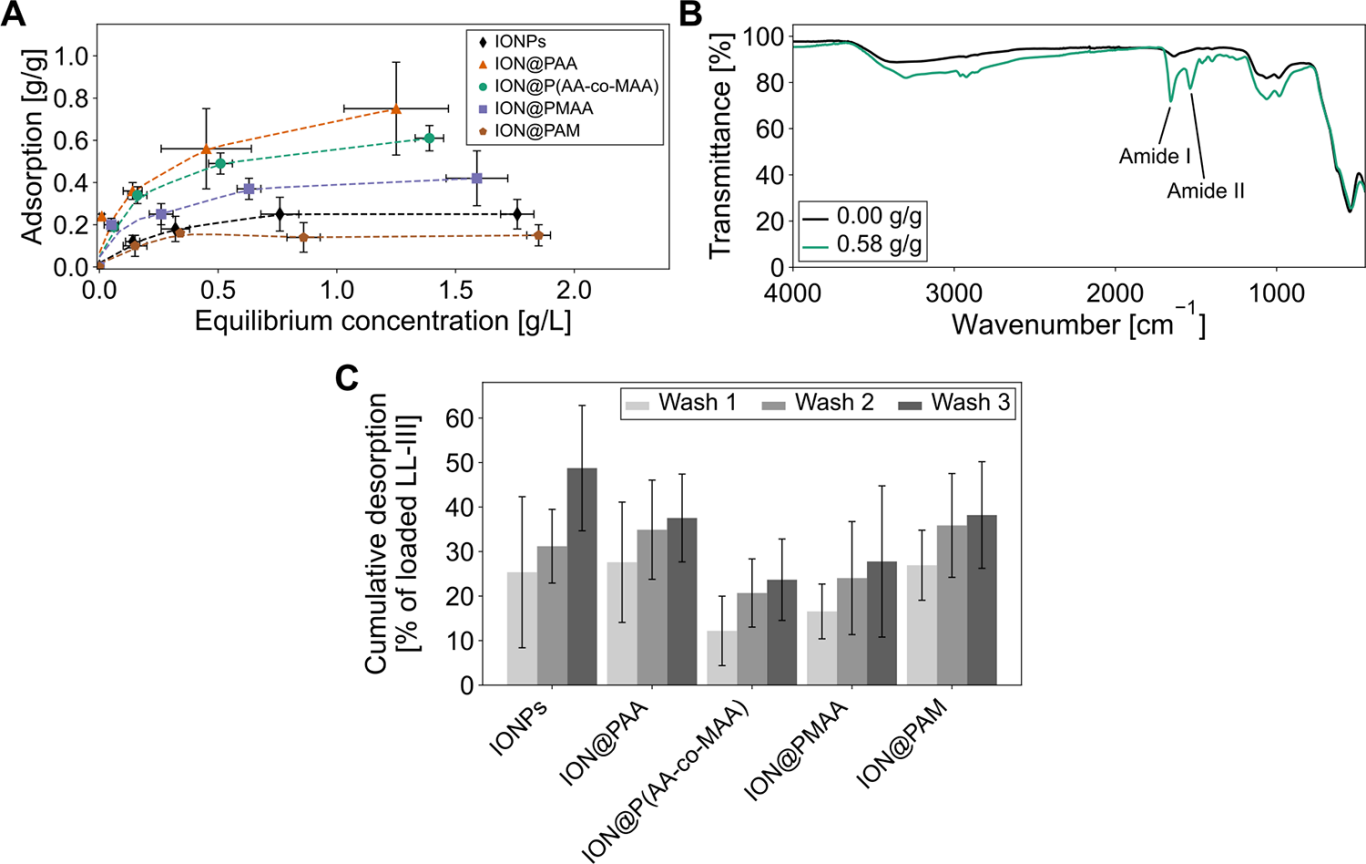
Comparison of the adsorption and desorption properties of bare IONPs, PAA-coated IONPs (ION@PAA), P(AA-*co*-MAA)-coated IONPs [ION@P(AA-*co*-MAA)], PMAA-coated IONPs (ION@PMAA), and PAM-coated (ION@PAM). (A) Adsorption of LL-III by bare and polymer-coated IONPs. (B) Representative ATR-FTIR spectra of ION@P(AA-*co*-MAA) without LL-III and with 0.52 g/g LL-III adsorbed (after washing step 3). (C) Cumulative desorption after the first, second, and third wash, expressed as percentage of the initially loaded LL-III (incubated with 2 g/L LL-III). Error bars indicate the standard deviation of three independent measurements in triplicates.

The adsorption and desorption properties of LL-III on various bare IONPs and polymer-coated particles were evaluated to determine the effect of the different polymer coatings on LL-III loading capacity and desorption properties, whereas the adsorption relies on noncovalent interactions between the particles and the peptide, exclusively. Initially, the adsorption of the peptide onto the particles was analyzed by ATR-FTIR which revealed the characteristic amide I and II bands at 1653 and 1537 cm^–1^ (Figures 2B and S2), respectively, on LL-III-loaded particles, indicating the noncovalent interaction between LL-III and the polymer coating.^39^

The maximum loading capacity of LL-III was found to vary significantly among the different nanoparticle formulations when incubated at the highest investigated LL-III concentration of 2 g/L (Figure 2A and Table 3). A comprehensive summary of these data is provided in Table S1. The uncoated IONPs exhibited an LL-III adsorption of 0.25 ± 0.08 g/g (Figure 2A and Table 3), which is consistent with the previously reported results^7^ and corresponds to a drug loading (DL) of 12%; whereas the drug loading was defined as the percentage of peptide adsorbed on the particles in terms of the initial amount of peptide used in the adsorption assay. Although electrostatic attraction between the positively charged LL-III and the positively charged IONPs, which is bridged by phosphate anions from the buffer, should facilitate adsorption, the presence of a hydration layer around the nanoparticles can reduce the effective surface charge and hinder strong interaction.^40^ Additionally, bare IONPs lack functional groups, such as carboxy groups, that could enhance strong binding with the peptide making the adsorption rely on relatively weak electrostatic forces, like hydrogen bonds.

**Table 3:** Summary of Particle Average Adsorption of LL-III after Incubation with 2 g/L LL-III (± Standard Deviation), Drug Loading, and Cumulative Desorption after Wash 3 for Each Particle System (±Standard Deviation) (Incubation with 2 g/L LL-III)^a^

particles	loading at 2 g/L LL-III [g/g]^b^	drug loading [%]^c^	average cumulative desorption after wash 3 [% of loaded LL-III]^d^
IONPs	0.25 ± 0.08	12	48.7 ± 14.1
ION@PAA	0.77 ± 0.24	38	37.5 ± 9.9
ION@P(AA*-co*-MAA)	0.61 ± 0.07	30	23.7 ± 9.1
ION@PMAA	0.42 ± 0.14	21	27.8 ± 17.0
ION@PAM	0.15 ± 0.06	7.5	38.2 ± 12.9

a Standard deviation was obtained by three independent measurements in triplicates.

b As determined by analysis of free LL-III in the supernatant by BCA assay.

c As calculated from peptide adsorption.

d Deduced from the peptide adsorption assay.

Overall, the polymer-coated IONPs could significantly enhance LL-III adsorption compared to bare IONPs (Figure 2A and Table 3) highlighting the crucial role that polymer functionalization plays in improving drug loading capacity. Among the different polymer-coated nanoparticles, ION@PAA demonstrated the highest LL-III adsorption (0.77 ± 0.24 g/g, DL = 38%), followed by ION@P(AA-*co*-MAA) with a slightly lower adsorption (0.61 ± 0.07 g/g, DL = 30%), and ION@PMAA with a more moderate loading of 0.42 ± 0.14 g/g (DL = 21%). This enhanced adsorption can be attributed to the presence of carboxy groups in these coatings (Figure 1E and Table 2), which likely facilitate stronger electrostatic interactions in addition to the formation of hydrogen bonds with the positively charged LL-III. The presence of aliphatic resiudes in the polymer composition was assessed by a semiquantitative ATR-FTIR analysis. Characteristic regions corresponding to aliphatic residues (3000–2800 cm^–1^) and carboxyl groups (1795–1345 cm^–1^) were compared with each other (Table S2) and revealed that the ratio of carboxyl to aliphatic groups was highest in PMAA-coated IONPs, followed by ION@P(AA-*co*-MAA), and lowest in ION@PAA (Figure 1E and Table S2). This difference in the polymer composition indicates the presence of methyl groups in PMAA- and P(AA-*co*-MAA)-coated IONs present which can interact with the hydrophobic core of the LL-III α helix,^41^ potentially enhancing the binding of LL-III even further.

The low variability of LL-III adsorption on P(AA-*co*-MAA) compared to PAA and PMAA (Figure 2A and Table 3) likely arises from the synergistic effect of combining AA and MAA monomers. The incorporation of MAA and thus an additional methyl group into the copolymer may result in a more uniform distribution of carboxylate groups, improving the accessibility of these functional sites for ion binding.^17,18^

While PAA has a higher density of carboxylate groups due to its lower molecular weight,^16^ the copolymer P(AA-*co*-MAA) still shows a more favorable ion exchange capacity due to the optimized interaction between the functional groups.^17,18^ In addition, although PMAA is known to be less flexible than PAA due to its higher glass transition temperature,^42^ the copolymer may provide improved structural stability and favorable adsorption properties. Since LL-III has an α-helical conformation,^28^ we hypothesize that the additional methyl group on the particles (Table S2) might improve the adsorption and the adsorption stability through hydrophobic effects between the methyl groups and the hydrophobic core.^41^ Thus, P(AA-*co*-MAA) provides adsorption with only low variability compared to the other polymer coatings, which is a crucial parameter for consistent and predictable drug loading on drug delivery devices.^43^

**Figure 3:**
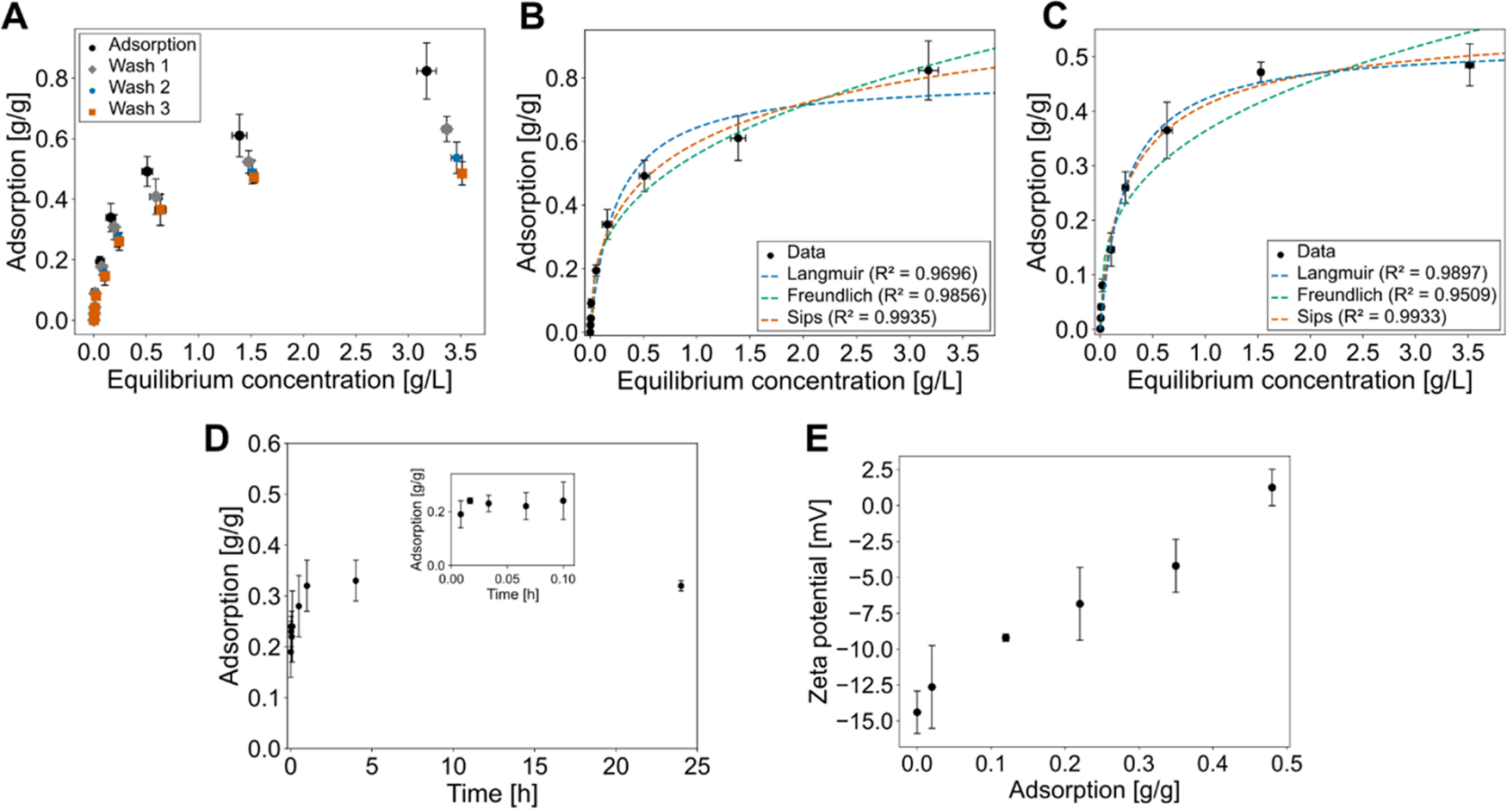
Adsorption isotherms of LL-III on P(AA-*co*-MAA)-coated IONPs [ION@P(AA-*co*-MAA)] and its effect on the ζ-potential. (A) Adsorption isotherm and desorption showing adsorption after incubation with the peptide, and after first, second, and third wash. Adsorption isotherm fitting for LL-III adsorption on ION@P(AA-*co*-MAA) (B) after incubation, and (C) after wash 3 with Langmuir, Freundlich, and Sips fit. (D) Adsorption kinetics of LL-III on ION@P(AA-*co*-MAA) over time, with a zoom-in representing durations up to 6 min (0.1 h). (E) ζ-potential of 50 mg/L ION@P(AA-*co*-MAA) loaded with varying amounts of LL-III in 50 mM PBS, pH 7.4. Error bars indicate the standard deviation of three independent measurements in triplicates.

For comparison, polyacrylamide-coated IONPs (ION@PAM) showed an adsorption of only 0.15 ± 0.06 g/g (Figure 2A and Table 3) corresponding to a DL of 7%. PAM coating was chosen as it is adsorbed on the surface of IONPs,^44^ like the AA- and MAA-based (co)polymers, and the amide group in PAM does not provide the same electrostatic interactions as the carboxy groups in the other polymer coatings. Thus, the absence of charged moieties resulted in weaker interactions with the cationic LL-III peptide, leading to a lower adsorption. However, it has been reported earlier that several factors, as prolonged storage and high temperatures, can lead to the hydrolysis of the amide, converting it into a carboxy group.^45^ The data derived from adsorption assays (Figure 3A and Table 3) and ATR-FTIR (Figure S3) suggests a low degree of hydrolysis making these particles a well-suited control for the anionic polymer-coated IONPs.

The standard deviation for LL-III adsorption on ION@PAA was remarkably high with ±0.24 g/g (Figure 2A and Table 3). This variability can likely be attributed to the properties of the PAA-coating and peptide-coating interactions. The PAA coating can undergo structural changes, depending on molecular weight and concentration, leading to heterogeneous peptide adsorption.^46^ Additionally, α-helical peptides may undergo conformational changes upon adsorption, exposing hydrophobic residues^47^ and causing aggregation of the peptide on the surface of the adsorbent.^48^ It is likely that this conformational change also occurs upon adsorption on ION@P(AA-*co*-MAA) and ION@PMAA. However, as shown earlier with bovine serum albumin and polymer-based nanoparticles, hydrophobic residues, such as methyl groups, can form robust interactions with hydrophobic domains of proteins,^41^ potentially stabilizing the peptide on the particles, thereby reducing variability in the adsorption process (Figure 2A and Table 3).

Taken together, these results emphasize the critical role of charge-based interactions and the nature of polymer coatings in promoting effective drug adsorption and optimizing nanoparticle-based drug delivery systems, as already suggested earlier.^26^ The carboxylate groups on the polymer coatings significantly modulate the interaction between nanoparticles and LL-III (Figure 2A and Table 3) enhancing electrostatic interactions and providing a favorable environment for hydrogen bonding with the peptide.^49^ This was especially evident when comparing uncoated IONPs and ION@PAM, both lacking functional groups for electrostatic peptide interactions, to PAA-, PMAA-, and P(AA-*co*-MAA)-coated nanoparticles. The introduction of polymer coatings containing carboxylate groups could enhance the adsorption of LL-III by 208% (ION@PAA), 144% [ION@P(AA-*co*-MAA)], and 68% (ION@PMAA) compared to bare IONPs.

The stability of the adsorption of LL-III on polymer-coated and bare IONPs was evaluated after performing three consecutive washing steps on the nanoparticles with PBS. The washing steps were conducted to determine the desorption resistance of the peptide on the nanoparticles. By repeatedly washing the particles, the amount of peptide remaining in solution indicates weak adsorption (when significant peptide is detected in solution) or strong and stable adsorption (when less peptide is detected in solution). This procedure helped to assess the strength and stability of peptide adsorption. The desorption was measured as a percentage of the initially adsorbed LL-III (Figure 2C and Table 3). Notably, the cumulative desorption of LL-III varied across the different particle systems, showing distinct patterns of binding strength and stability, which reflect the interactions between LL-III and the nanoparticle surfaces. More detailed desorption data are provided in Table S3.

The bare IONPs exhibited the highest desorption rate, with 48.7 ± 14.1% of the loaded peptide being released after three washes when incubated in 2 g/L LL-III (Figure 2C and Table 3) which is lower than previously reported for bare IONPs (63.6%).^7^ Still, the desorption is relatively high compared to the polymer-coated IONPs. As it has been shown previously, bare IONPs are predominantly positively charged at physiological pH.^7^ Phosphate anions from the buffer (PBS) can adsorb to the surface,^50^ bridging the cationic peptide to the IONP surface. Although electrostatic interactions between the IONPs, phosphate anions, and LL-III drive the adsorption of the peptide, our results (Figure 2C and Table 3) and a previous report^7^ suggest that the interactions are not as strong as compared to the interactions between the peptide and the anionic polymer-coated IONPs.

In contrast, ION@PAA demonstrated a lower desorption rate of 37.5 ± 9.86% (incubated at 2 g/L LL-III), despite having the highest LL-III adsorption on average (Figure 2C and Table 3). Interestingly, the high variability of the adsorption did not lead to a higher variability in the desorption of the peptide. This suggests that the carboxylate groups of the PAA coating, which are strongly negatively charged under physiological conditions,^51^ enhance the stability of the peptide adsorption, likely through electrostatic interactions between the negatively charged polymer and the positively charged LL-III peptide.^41^ The relatively stable interaction between the peptide and the PAA coating can prevent significant peptide loss during washing,^52^ resulting in lower desorption values compared to the bare nanoparticles.

ION@P(AA-*co*-MAA) showed the lowest desorption rate of 23.7 ± 9.14% (Figure 2C and Table 3) which may be due to the presence of both AA and MAA monomers in the copolymer. The combination of AA and MAA can facilitate a balance between carboxylates for strong electrostatic interactions and hydrophobicity provided by MAA which can interact with the hydrophobic residues of LL-III.^41^ The aliphatic index (162) and grand average of hydropathicity (0.373), which were obtained from ExPasy,^53^ revealed the hydrophobic profile of LL-III, supporting the hypothesis that hydrophobic interactions may slow down the desorption process. Thus, we hypothesize that this results in stronger binding interactions with LL-III leading to a stabler adsorption and reduction of peptide desorption. Also, ION@PMAA exhibited a low desorption rate of 27.8 ± 17.0% which supports the hypothesis that the adsorbed peptide is stabilized on the particle surface by the combination of electrostatic interaction and hydrophobic effect.

ION@PAM showed intermediate desorption values of 35.3 ± 17.5% (Figure 2C and Table 3), reflecting the overall low peptide adsorption on these particles. Since the PAM coating can form hydrogen bonds with the peptide,^54^ the peptide is not completely desorbed after three washing steps. However, due to the absence of strong electrostatic interactions, the adsorption of LL-III onto ION@PAM is much weaker compared to the particles with an anionic polymer coating.

In general, the desorption data indicate that the stability and strength of peptide adsorption is influenced by the polymer coating composition. PAA coating promotes a high adsorption with moderate desorption while PMAA provides a lower but more robust adsorption of LL-III (Figure 2A,C and Table 3). By combining both monomers, AA and MAA, in P(AA-*co*-MAA) coating, LL-III adsorption was enhanced compared to PMAA and desorption was reduced compared to PAA. (Figure 2A,C and Table 3). These favorable properties are most likely due to the balance between carboxylates and hydrophobic methyl residues interacting with the cationic residues and the hydrophobic core of LL-III, respectively.^28,41^ ION@PAM showed a moderate desorption, possibly due to overall low LL-III adsorption which is stabilized by hydrogen bonding. These results underscore the importance of polymer structure and functional groups in modulating peptide adsorption and desorption dynamics,^55^ which is vital for optimizing nanoparticle-based drug delivery systems and other biomedical applications.

Overall, ION@P(AA-*co*-MAA) showed the most promising characteristics for a drug delivery system with a small size, good and stable adsorption of LL-III with low variability, which indicates a reduction of drug leakage in potential applications for drug delivery.

### Adsorption Dynamics and Characteristics of LL-III on Poly(acrylic-acid-*co*-methacrylic acid)-Coated Iron Oxide Nanoparticles

ION@P(AA-*co*-MAA) were selected for further investigation due to their favorable properties for drug delivery, including particle size, LL-III adsorption, and adsorption stability. Despite lower LL-III adsorption than PAA-coated particles, ION@P(AA-*co*-MAA) showed an overall lower variability, and low peptide desorption after multiple washes, making them a promising option for sustained drug release and reduction of drug leakage. Thus, we decided to investigate the adsorption dynamics and kinetics of LL-III on ION@P(AA-*co*-MAA) in more detail.

Figure 3A presents the adsorption isotherm of LL-III on ION@P(AA-*co*-MAA), highlighting both the initial loading after incubation and the desorption following multiple washes. As expected, LL-III adsorption increased with the peptide concentration, reaching its maximum at the highest investigated peptide concentration (4 g/L) with 0.82 g/g (Figure 3A and Table S4). The desorption was minimal, with approximately 63% of the peptide retained on the nanoparticles (4 g/L) after the first wash, and 49% after three washes (4 g/L) (Figure 3A and Table S4). These results indicate a stable adsorption with low desorption suggesting that ION@P(AA-*co*-MAA) provides an ideal platform for drug delivery, offering high loading capacity and minimal drug leakage under physiological conditions thereby potentially minimizing off-target effects of the drug.

To get a better understanding of the underlying adsorption mechanisms, the adsorption data for ION@P(AA-*co*-MAA) were fitted to Langmuir, Freundlich, and Sips isotherms (Figure 3A,B). Each model highlights different aspects of the system: the Langmuir fit assumes monolayer adsorption on homogeneous surfaces, while the Freundlich fit accounts for surface and adsorption heterogeneity, and Sips integrates elements of both Langmuir and Freundlich, accommodating systems with mixed adsorption mechanisms.^56^ By evaluating these models, we wanted to gain insights into the surface uniformity, and whether the process involves monolayer, multilayer, and/or heterogeneous interactions helping to reveal the driving forces and efficiency of adsorption in this particle system.

The isotherm fitting results showed that the Sips model, accounting for both monolayer and heterogeneous adsorption, provided the best fit (Figure 3B, *R*^2^ = 0.9935). This indicates that LL-III adsorption on ION@P(AA-*co*-MAA) involves a combination of adsorption mechanisms.^56^ After the third wash of the particles (Figure 3C), the Sips model still provided the highest *R*^2^ value (0.9933), confirming heterogeneous binding sites with varying stability.^56,57^ A heterogeneous surface was expected due to the characteristics of the P(AA-*co*-MAA) coating, likely arising from factors such as the polymer concentration on the surface, polymer–nanoparticle surface interactions, and coating density which create regions with different charge densities.^58,59^

The kinetics of LL-III adsorption on ION@P(AA-*co*-MAA) showed a rapid adsorption, with significant peptide adsorption already within the first 10 min with an adsorption of 0.19 g/g (Figure 3D and Table S5). A closer investigation of the first 6 min showed that the initial rapid loading phase accounts for most of the adsorption (close-up in Figure 3D). After 1 h, the system approached equilibrium and after 4 h, the system reached an equilibrium loading of 0.31 g/g without changing considerably over 24 h. A similar rapid adsorption was observed for bare IONPs which also showed a fast adsorption of the peptide within the first 5 min and reaching equilibrium already after 30 min.^7^ These results suggest that the nanoparticles can efficiently load a cationic drug in a short time which is beneficial, in particular, when handling drugs with low physicochemical stability.

The colloidal stability and swelling behavior of ION@P(AA-*co*-MAA) were assessed by measuring ζ-potential and hydrodynamic diameter of the particles across pH 2 to 12. As expected, the nanoparticles exhibited a highly negative ζ-potential in water, ranging from −35 to −41 mV (Figure S4) between pH 5 and 12, which aligns with previously reported results.^10^ The negative ζ-potential increased gradually starting between pH 5 and 4.5, which corresponds to the p*K*_a_ range of the carboxy groups on the polymer coating.^60^ Equally, the particle size increased significantly between pH 4 and 4.5 (Figure S4), suggesting particle aggregation at lower pH values. At pH 2, the particles had a hydrodynamic diameter of 1 μm, indicating a loss of stability in highly acidic conditions, most likely due to the lack of sufficient surface charge to maintain colloidal stability.^61^ Between pH 4.5 and 12, however, the particle size remained stable, which is optimal for drug delivery, as it ensures that the nanoparticles maintain their colloidal stability and can circulate effectively in the bloodstream.^62^

The ζ-potential was also measured under varying LL-III loading concentrations to assess the impact of peptide adsorption on the particle surface charge (Figure 3E). Initially, ION@P(AA-*co*-MAA) had a ζ-potential of −14.4 mV in 50 mM PBS at pH 7.4, showing a negatively charged surface. The shift toward a less negative ζ-potential in PBS, compared to water, can be explained by the ionic strength, which causes ion screening and compresses the electrical double layer around the nanoparticles reducing electrostatic repulsion between the particles and leading to a lower surface charge.^63^ As LL-III adsorption increased, the ζ-potential became less negative, reflecting the increasing cationic nature of the peptide. At the highest investigated concentration of adsorbed LL-III (0.6 g/g), the ζ-potential shifted to +2.1 mV, suggesting that the adsorption of the positively charged peptide alters the surface charge of the nanoparticles. This is in line with previous results reporting an increase in ζ-potential with an increase in LL-III loading,^7^ highlighting the role of electrostatic interactions in peptide adsorption on ION@P(AA-*co*-MAA).^64^

**Figure 4:**
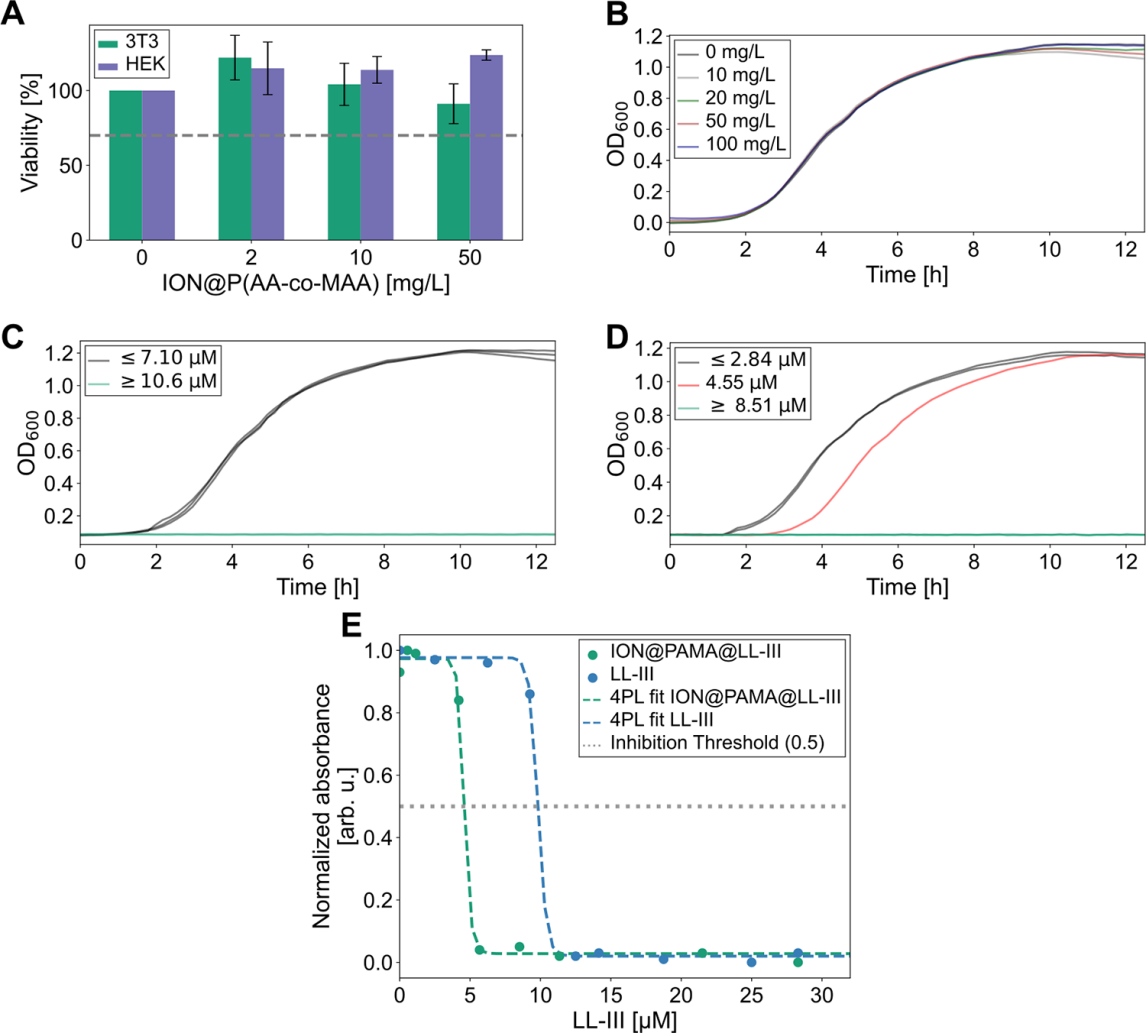
Cytotoxicity studies of LL-III-loaded ION@P(AA-*co*-MAA). (A) Viability of 3T3 and HEK cells incubated with the indicated concentration of ION@P(AA-*co*-MAA) and viability threshold at 70% (dashed line) Error bars indicate the standard deviation of the measurement in triplicates. Mean growing curve of *Escherichia coli* cells over time incubated with varying concentration of (B) ION@P(AA-*co*-MAA), (C) LL-III, and (D) LL-III when loaded on ION@P(AA-*co*-MAA) obtained in three independent experiments in duplicates. (E) Normalized absorbance of *E. coli* incubated for 4.5 h with the indicated concentration of LL-III loaded on ION@P(AA-*co*-MAA) or free LL-III performed in triplicates with the respective four parameter logistic regression and inhibition threshold of 0.5 (dashed gray line).

ION@P(AA-*co*-MAA) nanoparticles proved favorable properties for drug delivery, including efficient and stable LL-III adsorption (Figures 2A and 3A), low desorption (Figures 2C and 4A), and rapid loading kinetics (Figure 3D). The Sips adsorption model provided the best fit for the isotherm data (Figure 3B,C), indicating that adsorption occurs on both monolayer and heterogeneous sites.^58,59^ The particles also show a positive shift in ζ-potential upon LL-III loading (Figure 3E), reflecting the electrostatic nature of the interaction between the peptide and the nanoparticle surface.^64^ Importantly, the nanoparticles maintain colloidal stability at physiological pH (Figure S4), making them well-suited for drug delivery applications. Overall, the ION@P(AA-*co*-MAA) system shows significant promise as a stable and efficient drug delivery platform for targeted therapy.

### Cytotoxic Effects of ION@P(AA-*co*-MAA) and LL-III

The cytotoxicity of ION@P(AA-*co*-MAA) was evaluated in vitro in 3T3 fibroblasts, HEK cells, and *E. coli* to assess the nanoparticle’s safety and antimicrobial activity, focusing on the effects of LL-III loading on cytotoxic effects and inhibition of bacterial growth.

The cytotoxicity was first assessed in the mammalian cell lines to evaluate the safety of ION@P(AA-*co*-MAA). As shown in Figure 4A, cell viability remained above 70% for both 3T3 and HEK cells at various nanoparticle concentrations, indicating noncytotoxicity. Additionally, LL-III-loaded nanoparticles had no negative impact on cell viability (Figures S5A,B, and S6A–C) either. The cytotoxicity remained low across a range of adsorbed LL-III concentrations, with no significant difference in viability for either cell line at any tested peptide-loaded nanoparticle concentration. However, a cytotoxic effect was observed between 0.566 and 5.66 μM when LL-III was applied to the cells without loading it on ION@P(AA-*co*-MAA) (Figure S5C), consistent with previous studies.^27,65^ These results suggest that ION@P(AA-*co*-MAA) are biocompatible with mammalian cells, even when loaded with the highest tested LL-III concentration [19 μM LL-III adsorbed on ION@P(AA-*co*-MAA)]. This may be due to the mechanism of the adsorption of the peptide. As the adsorption is mediated by electrostatic interactions, it could alter its conformation^17,47^ and reduce the ability for cell membrane disruption. Thus, the polymer coating may shield the peptide from interacting with the cellular membrane,^66^ preventing it from adopting a conformation required for integrating into the cellular membrane.

The antimicrobial activity of ION@P(AA-*co*-MAA), LL-III, and peptide-loaded particles was assessed in *E. coli*. ION@P(AA-*co*-MAA) alone did not show any antimicrobial activity (Figure 4B), similar to the results obtained in the mammalian cells (Figure 4A). As expected, LL-III demonstrated antimicrobial activity (Figure 4C), but only at concentrations above 9.25 μM (Figure 4E), higher than reported in earlier studies (1.4–3.7 μM).^7,27^ This difference may be due to variations in *E. coli* strains, antibiotic resistance, or the growth medium used.^67^

Notably, LL-III-loaded ION@P(AA-*co*-MAA) were able to inhibit *E. coli* growth at much lower peptide concentrations (Figures 4D and S7). At 4.55 μM, bacterial growth noticeably slowed, and at 8.51 μM, no growth could be observed, indicating an enhanced antimicrobial activity of LL-III when loaded on the particles. The minimum inhibitory concentration (MIC) of LL-III was modeled from the optical density of *E. coli* after 4.5 h of incubation (log-phase growth) using a four-parameter logistic regression (4PL fit), with the threshold for inhibition set at 50% of bacterial growth reduction which corresponds to a normalized optical density of 0.5 (Figure 4E). This method allowed us to identify the concentration at which the peptide effectively inhibited bacterial growth significantly. The MIC for LL-III decreased from 9.82 μM (free LL-III) to 4.59 μM when loaded on the nanoparticles (Figure 4E). This suggests that the adsorption on the nanoparticles could lead to a local accumulation of the peptide at the bacteria membrane, enhancing the antimicrobial activity of LL-III.

In conclusion, the LL-III-loaded ION@P(AA-*co*-MAA) showed excellent biocompatibility in mammalian cells (Figure 4A) and the peptide-loaded nanoparticles exhibited enhanced antimicrobial activity against *E. coli*, with a dramatic reduction in MIC (Figure 4D,E). These results suggest that the nanoparticles are a stable, nontoxic delivery platform for LL-III, that enhances its antimicrobial efficacy, making them a promising candidate for both drug delivery and antimicrobial applications without cytotoxic effects for mammalian cells.

## Conclusions

Our study highlights the potential of ION@P(AA-*co*-MAA) as a platform for drug delivery of cationic drugs and antimicrobial applications, offering superior adsorption efficiency, stability, and antimicrobial potency compared to existing nanoparticle systems.^7,8,68^ Coating IONPs with P(AA-*co*-MAA) enhanced the adsorption of the cationic peptide LL-III (Figure 3A) with an adsorption of 0.62 g/g (DL = 30%) when incubated with 2 g/L LL-III, compared to bare IONPs (0.25–0.35 g/g, DL = 22%),^7^ silica-coated IONPs (0.28 g/g, DL = 14%),^8^ and carboxymethyl dextran-coated IONPs (0.32 g/g, DL = 24%),^68^ under the same conditions. The adsorption is characterized by rapid kinetics (Figure 3D) and high loading efficiency (Figure 3A), with significant adsorption within 10 min (close-up in Figure 3D).

The enhanced adsorption can be attributed to the customizable polymer coating, which provides strong electrostatic interactions combined with hydrophobic effects, resulting in an efficient and stable peptide adsorption.^26^ The process is best described by the Sips model (Figure 3B,C) indicating the heterogeneous binding sites.^56,57^ The nanoparticles demonstrated superior desorption resistance, retaining an average of 23.7% of adsorbed LL-III after three washes (Figure 2C and Table 3), especially when compared to other IONP-based delivery systems for LL-III. These systems showed over 50% desorption (bare IONPs)^7^ and over 90% desorption (carboxymethyl dextran-coated IONPs),^68^ resulting in a significant loss of the initially adsorbed peptide. A similarly low adsorption could only be achieved using silica-coated IONPs, which retained 75% of the peptide after washing.^8^ Overall, this high adsorption and low desorption of LL-III to/from ION@P(AA-*co*-MAA) underscore the potential of customized polymer coatings for targeted drug delivery with minimal drug leakage.

ION@P(AA-*co*-MAA) showed excellent biocompatibility when applied to 3T3 fibroblasts and HEK cells (Figure 4A), even when loaded with LL-III (5A, B). The polymer coating effectively shielded LL-III, reducing its cytotoxic effects on mammalian cells and maintained robust colloidal stability across a broad pH range (Figure S4), supporting their potential for biological use.^62^

Importantly, our study showed that LL-III-loaded ION@P(AA-*co*-MAA) significantly enhanced the antimicrobial activity of the peptide, reducing the MIC from 9.82 μM (free LL-III) to 4.59 μM when adsorbed on the particles (Figure 4E), suggesting that the system facilitates more effective delivery to bacterial membranes. This enhancement likely results from the ability of the nanoparticles to concentrate the peptide at the bacterial membrane and synergistically enhance membrane disruption. The improved efficacy at lower peptide concentrations highlights the potential of the nanoparticles to address the growing challenge of antimicrobial resistance without causing cytotoxicity in mammalian cells (Figure 4A).

These findings have broad implications for the development of advanced drug delivery systems. The robust adsorption and desorption properties of ION@P(AA-*co*-MAA), combined with their high biocompatibility, position these nanoparticles as an effective and adaptable platform for the delivery of cationic peptides and other therapeutic agents. Their ability to enhance antimicrobial efficacy while minimizing cytotoxic effects on mammalian cells supports their potential use in treating infections, particularly those involving multidrug-resistant bacteria. Additionally, the customizable polymer coating enables applications beyond antimicrobial therapies, such as targeted drug delivery in cancer treatment and precision medicine.

Overall, this study establishes ION@P(AA-*co*-MAA) as a versatile drug delivery system, with a strong potential for future *in vivo* studies to evaluate biodistribution, pharmacokinetics, and therapeutic efficacy. Their ability to enhance therapeutic outcomes while preserving biocompatibility is their potential to address current challenges in both antimicrobial therapy and broader drug delivery applications.

## Experimental Section

### Materials

Acrylic acid (≥99%, stabilized with hydroquinone monomethyl ether), hydrochloric acid (37%), iron(II)chloride tetrahydrate (98%), iron(III)chloride anhydrous (97%), methacrylic acid (≥99%, stabilized with hydroquinone monomethyl ether), sodium dodecyl sulfate (≥99%,), sodium hydroxide pellets (≥97%), sodium phosphate dibasic anhydrous (≥99%), and sodium phosphate monobasic anhydrous (≥98%) were purchased from Sigma-Aldrich Handels GmbH (Vienna, Austria). Acrylamide (≥99%), and ammonium persulfate (≥98%) were purchased from Carl Roth (Karlsruhe, Germany). Lasioglossin-III peptide (VNWKKILGKIIKVVK) was purchased from GL Biochem Ltd. (Shanghai, China), and GenScript Biotech BV (Rijswijk, Netherlands).

### Synthesis of Iron Oxide Nanoparticles

Superparamagnetic iron oxide nanoparticles (IONPs) were prepared by coprecipitation in accordance with a previously established and published protocol.^7^ For the synthesis, 14.45 g (361.5 mmol) sodium hydroxide was dissolved in 200 mL degassed ultrapure water. 10.4 g (64 mmol) anhydrous FeCl_3_ and 7.0 g (35.2 mmol) FeCl_2_ tetrahydrate were dissolved in 80 mL degassed ultrapure water. The iron salts were added slowly to the sodium hydroxide solution under continuous mechanical stirring, and the reaction was allowed to continue for 30 min. The resulting particles (IONPs) were transferred to a glass flask and washed 15 times with degassed ultrapure water by magnetic decantation until a pH of ≥7.4 was reached. Thereafter, the IONPs were resuspended in 200 mL degassed ultrapure water and stored at 4 °C. The mass concentration was determined gravimetrically by drying the particles overnight at 60 °C.

### Polymer Coating of Iron Oxide Nanoparticles

The polymer coating of IONPs was performed based on two adapted and modified protocols.^10,13^ 100 mg IONPs were placed in a glass flask with a ported cap and filled up to 100 mL with degassed ultrapure water. The suspension was redispersed using an ultrasonic processor (Model 120 Sonic Dismembrator, Fisherbrand) set to 75% amplitude. Subsequently, the suspension was transferred to an ultrasound bath and connected to a vacuum source for degassing for 30 min. The bottle was evacuated with nitrogen and bubbled with nitrogen for a further 20 min. Under continuous stirring (250 rpm), 575 mg (2 mmol) sodium dodecyl sulfate was added, and the suspension was heated to 70 °C in a water bath. Once the temperature of 70 °C had been reached, the respective monomers were added. For poly(acrylic acid)-coated IONPs (ION@PAA), a total of 411.8 μL (6 mmol) acrylic acid were used for the coating. For poly(acrylic acid-*co*-methacrylic acid)-coated IONPs [ION@P(AA-*co*-MAA)], a total of 205.9 μL (3 mmol) acrylic acid and 253.1 μL (3 mmol) methacrylic acid were used for the coating. For poly(methacrylic acid)-coated IONPs (ION@PMAA), 506.2 μL (6 mmol) methacrylic acid were used for the coating. As a control, poly(acrylamide)-coated IONPs (ION@PAM) were prepared using 426.5 mg (6 mmol) acrylamide. Following the addition of the monomers, the suspension was left to equilibrate for a period of 45 min. Then, 825 mg (3.61 mmol) ammonium persulfate (APS) was dissolved in 4 mL ultrapure water and added to the suspension to initiate the polymerization process. The reaction was kept at a constant temperature of 70 °C with continuous stirring. Throughout the synthesis, the headspace in the reaction vessel was purged with nitrogen. Following the polymerization, the polymer-coated nanoparticles were separated magnetically and washed with 2 L ultrapure water. After washing, the nanoparticles were redispersed in ultrapure water and stored at 4 °C. The mass concentration was determined gravimetrically by drying the particles at 60 °C overnight. ION@PAM were used within 7 days after synthesis to limit hydrolysis of the amide residues to carboxylic acid.^45^

### Physicochemical Characterization of Bare and Polymer-Coated Iron Oxide Nanoparticles

The hydrodynamic diameter of the nanoparticles was determined by dynamic light scattering (VASCO Flex, Cordouan Technologies SAS). To this end, the respective nanoparticle system was diluted with ultrapure water (pH ≈ 7.2) to a concentration of 50 mg/L. The samples were placed in disposable plastic cuvettes (Brand GmbH + CO KG) and analyzed at room temperature. For the determination of the ζ-potential and the hydrodynamic diameter across a pH range (2 to 12), a particle suspension was initially adjusted to pH 12 using NaOH. The pH was then gradually lowered with HCl, and samples were collected at the indicated pH values for analysis. Finally, the nanoparticles were brought to a final concentration of 50 mg/L using the respective solvent and analyzed at 25 °C using a Zetasizer Nano ZS (Malvern Panalytical, Ltd.).

#### ATR-FTIR

For analysis of the chemical composition using ATR-FTIR, 1 μL of nanoparticle suspension (2 g/L) was placed on the ATR crystal and the liquid was evaporated by the application of cold air. The data were recorded (32 scans) using a UATR-FTIR (Spectrum Two, PerkinElmer, Inc.) equipped with a diamond ATR crystal and DTGS detector at room temperature. The integral of characteristic regions for aliphatic residues (3000–2800 cm^–1^) and carboxy groups (1795–1345 cm^–1^) were calculated with the spectroscopy software Spectragryph.^69^

#### TEM

Morphology and size of the IONPs was evaluated using a transmission electron microscope (Tecnai G20, FEI Company), operated at a voltage of 120 kV. The nanoparticles were diluted to a concentration of 10 mg/L with ultrapure water and redispersed using an ultrasonic processor set to 75% amplitude. The suspension was applied to carbon-coated copper grids (200 mesh, PELCO), which had been glow discharged in advance (PELCO easiGlow, Ted Pella, Inc.). Images were acquired using a CCD camera (BM-Ultrascan 1000P, Gatan, Inc.). The particle size was analyzed in Fiji.^70^

#### VSM

Magnetic measurements were performed using vibrating sample magnetometer (Lake Shore Cryotronics, Inc.) with an EM7-CSB magnet with a maximum magnetic field strength of 3.2 T. Samples were freeze-dried before measurement. Measurements were taken over 9 field segments, starting at 0 Oe, with continuous data acquisition at 293 K.

#### XPS

IONPs were freeze-dried for XRD analysis. Measurements were performed with an XPS system with dual anode (Al, Mg) X-ray source (XR50, SPECS Surface Nano Analysis GmbH) and a hemispheric analyzer (Phoibos 150, SPECS Surface Nano Analysis GmbH). Photoelectrons were excited by a non-monochromatized AlKα radiation (400 W) and detected at an angle perpendicular to the sample surface. A pass energy of 50 eV was selected for the measurements. Calibration was performed using an internal standard, aliphatic carbon, by setting the peak of the C 1s line to 285.0 eV.

#### XRD

IONPs were freeze-dried for XRD analysis. Measurements were carried out using a STADI P diffractometer (STOE & Cie GmbH) equipped with a molybdenum X-ray source with a wavelength of 0.709 Å.

### Adsorption Isotherms

The evaluation of the adsorption capacity was performed based on an adapted protocol published earlier.^7^ For the initial comparison between the three different polymer-coated IONP systems, varying LL-III concentrations (final LL-III concentrations: 0, 0.25, 0.5, 1, 2 g/L) were mixed with 2 g/L of the respective particles, ION@PAA, ION@P(AA-*co*-MAA), or ION@PMAA (final concentration: 1 g/L). For the determination of the adsorption isotherm of ION@P(AA-*co*-MAA), in total eight LL-III concentrations (final LL-III concentrations: 0, 0.025, 0.05, 0.1, 0.25, 0.5, 1, 2, 4 g/L) were evaluated. In all experimental conditions, LL-III was dissolved in 0.1 M PBS (pH 7.4) and diluted to the respective concentrations. The peptide solution was mixed with the corresponding polymer-coated ION stock (2 g/L in ultrapure water) in an equivalent ratio and incubated on an orbital shaker at 1000 rpm and 24 °C for 6 h. Afterward, the particles were separated magnetically for 10 min, and 200 μL of the supernatant was collected for subsequent analysis. To wash the particles, the remaining supernatant was discarded, and the polymer-coated nanoparticles were resuspended in 1 mL 50 mM PBS. The particles were incubated on an orbital shaker at 1000 rpm and 24 °C for 10 min and then separated magnetically. A total of 200 μL supernatant was collected (wash 1) for analysis, and the washing procedure was repeated two more times (washes 2 and 3). 25 μL of each sample was placed in a 96-well plate, and the total peptide concentration was analyzed using the Pierce BCA Protein Assay Kit (Thermo Fisher Scientific GmbH). For the standard curve, different concentrations of LL-III were prepared in 50 mM PBS. The concentration was determined by spectrophotometry at a wavelength of 568 nm using an ultraviolet–visible (UV–vis) spectrophotometer (PowerWave Select X, Bio-Tek Instruments, Inc.). The amount of LL-III adsorbed onto the surface of the particles was calculated by subtracting the mass of the free peptide in the supernatant from the initial peptide mass. The drug loading was defined as the percentage of peptide adsorbed on the particles in terms of the initial amount of peptide used for the adsorption assay. The desorption during the washing steps was measured, along with the cumulative desorption expressed as a percentage. Additionally, the adsorption of LL-III on the particle surface was analyzed by determination of the ζ-potential and ATR-FTIR of the particles with and without loading. For the analysis of the ζ-potential and adsorption, LL-III-loaded nanoparticles were used after being washed 3 times. The analysis by ATR-FTIR was performed with particle samples collected from the different washing stages. Both analyses were performed according to the procedures already mentioned in the Physicochemical Characterization of Bare and Polymer-Coated Iron Oxide Nanoparticles section.

### Adsorption Kinetics

To determine the kinetics of LL-III adsorption onto the particle surface, LL-III was prepared as a 1 g/L solution in 0.1 M PBS, pH 7.4, and ION@P(AA-*co*-MAA) as a 2 g/L suspension with ultrapure water. Peptide solution and particle suspension were mixed in a 1:1 ratio to obtain a total volume of 500 μL and incubated on an orbital shaker for the indicated duration at 24 °C and 1000 rpm. Afterward, 100 μL supernatant was collected by magnetic separation for 10 min and subsequently analyzed using the Pierce BCA Protein Assay Kit (Thermo Fisher Scientific GmbH) as described before.

### Cytotoxicity Assay in Mammalian Cells

The cytotoxicity of cells was verified with an XTT assay (SERVA GmbH). Both HEK-blue and 3T3 cells have been tested. The assay was prepared according to the guidelines of SERVA GmbH. Cells at a concentration between 105 and 106 cells in 100 μL were incubated in serum at 37 °C in a CO2 incubator for 48 h. 50 μL of the reconstituted XTT mixture was added to each well and mixed before incubation for 2 h. Absorbance at 450–500 nm was measured and evaluated with a UV–vis spectrophotometer (PowerWave Select X, Bio-Tek Instruments, Inc.).

### Antimicrobial Activity in *E. coli*

LL-III was prepared as a 4 g/L solution in filtered (0.22 μm) 0.1 M PBS (pH 7.4). ION@P(AA-*co*-MAA) were prepared as a 2 g/L suspension in autoclaved ultrapure water. For the loading of LL-III, an adsorption experiment was performed (24 °C, 6 h, 1000 rpm) with one subsequent washing step as described for adsorption isotherms. Additionally, different dilutions of LL-III and ION@P(AA-*co*-MAA) were prepared to assess the respective antimicrobial effect separately. *E. coli* BL21 (DE3) with a resistance against kanamycin was cultured overnight in Luria–Bertani broth with 50 mg/L kanamycin at 37 °C in an orbital shaker incubator. On the next day, the overnight culture was diluted to OD_600_ 0.01. On a sterile 96-well plate, 180 μL of the diluted *E. coli* culture was mixed with 20 μL of ION@P(AA-*co*-MAA), LL-III, or LL-III-loaded ION@P(AA-*co*-MAA). Additionally, each sample was mixed with 180 μL culture medium without bacteria to subtract the influence on the OD_600_ from the different amounts of particles and LL-III. The plate was incubated at 37 °C in a UV–vis spectrophotometer (PowerWave Select X, Bio-Tek Instruments, Inc.) with continuous shaking. Measurements were taken every 10 min at a wavelength of 600 nm. The minimum inhibitory concentration was modeled using a four-parameter logistic regression for OD_600_ measurements (normalized) after 4.5 h of incubation. The threshold for growth inhibition was set to 50% reduction of bacterial growth, corresponding to a normalized optical density of 0.5.

#### Drop Assay

Bacterial cultures incubated overnight with ION@P(AA-*co*-MAA), LL-III, or LL-III-loaded ION@P(AA-*co*-MAA) were each applied to an LB agar plate with kanamycin by adding 10 μL drops. The plates were then incubated at 37 °C overnight. Bacterial growth was assessed by optical observation the following day.

### Data Analysis and Visualization

The data was analyzed and visualized in Python 3.12.0. For data analysis, the SciPy^71^ and Scikit-learn^72^ packages were used, while data visualization was performed with the Matplotlib^73^ package.

## Supporting Information Available

ATR-FTIR spectra; hydrodynamic and ζ-potential measurements; cytotoxicity data; and adsorption and desorption data for nanoparticles (PDF)

## Abbreviations

4PL: four-parameter logistic
AA: acrylic acid
ATR-FTIR: attenuated total reflectance Fourier-transform infrared spectroscopy
DL: drug loading
DLS: dynamic light scattering
FDA: food and drug administration
HEK: human embryonic kidney cells
IONPs: iron oxide nanoparticles
ION@PAA: poly(acrylic acid)-coated iron oxide nanoparticles
ION@PAA-*co*-MAA: poly(acrylic acid-*co*-methacrylic acid)-coated iron oxide nanoparticles
ION@PAM: polyacrylamide-coated iron oxide nanoparticles
ION@PMAA: poly(methacrylic acid)-coated iron oxide nanoparticles
ION@P(AA-*co*-MAA): poly(acrylic acid-*co*-methacrylic acid)-coated iron oxide nanoparticles
LL-III: lasioglossin-III
MAA: methacrylic acid
MIC: minimum inhibitory concentration
MPI: magnetic particle imaging
PAA: poly(acrylic acid)
PAA-*co*-MAA: poly(acrylic acid-*co*-methacrylic acid)
PAM: polyacrylamide
PBS: phosphate-buffered saline
PMAA: poly(methacrylic acid)
P(AA-*co*-MAA): poly(acrylic acid-*co*-methacrylic acid)
SDS: sodium dodecyl sulfate
TEM: transmission electron microscopy
VSM: vibrating sample magnetometry
XRD: powder X-ray diffraction
XPS: X-ray photoelectron spectroscopy
