# Supplementary material for: Biocompatible Poly(acrylic acid-co-methacrylic acid)-Coated Iron Oxide Nanoparticles for Enhanced Adsorption and Antimicrobial Activity of Lasioglossin-III
Source: ACS Appl Mater Interfaces. 2025 Mar 5;17(11):16644–57. doi: 10.1021/acsami.4c22603 (PMC11931491; doi:10.1021/acsami.4c22603)
Supplement: Supplementary file 1 — am4c22603_si_001.pdf [file am4c22603_si_001.pdf]

Supporting Information

Biocompatible Poly(acrylic acid-co-methacrylic acid)-Coated Iron Oxide Nanoparticles for Enhanced Adsorption and Antimicrobial Activity of Lasioglossin-III

*AUTHOR NAMES. Marco Reindl[1], Verena Zach[1], Sebastian P. Schwaminger[1,2,3]**

AUTHOR ADDRESS. 1 NanoLab, Division of Medicinal Chemistry, Otto Loewi Research Center, Medical University of Graz, Neue Stiftingtalstraße 6, 8020 Graz, Austria.

2 Bioseparation Engineering Group, Department of Mechanical Engineering, Technical University of Munich, Boltzmannstraße 153, 85748 Garching, Germany.

3 BioTechMed-Graz, Mozartgasse 12/II 8010 Graz, Austria.

Corresponding Author

*Sebastian P. Schwaminger. E-mail: s.schwaminger@tum.de; sebastian.schwaminger@medunigraz.at. Phone: +43 316 38572125.

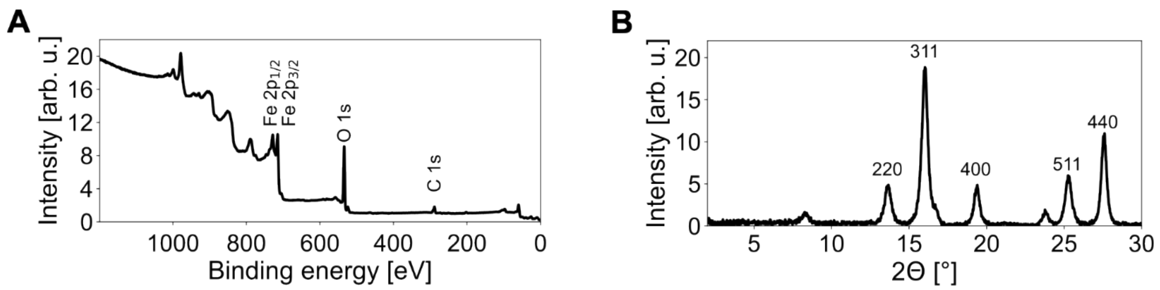

Figure S1. Characterization of bare iron oxide nanoparticles (IONPs). (A) XP spectra and (B) XRD pattern with relevant peaks indicated.

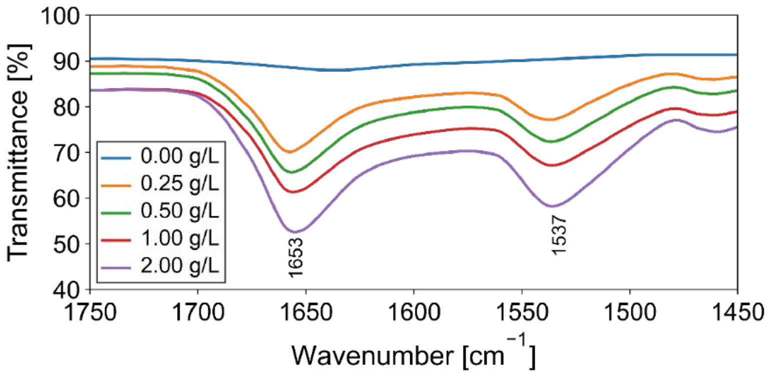

Figure S2. Representative ATR-FTIR spectra of ION@P(AA-co-MAA) particles loaded with LL-III after three washing steps indicating the presence of amide I (1653 cm^{-1}) and II (1537 cm^{-1}) on the particles. LL-III concentration given as initial LL-III concentration for incubation.

Table S1. Mean adsorption and equilibrium concentration of LL-III by bare and polymer-coated IONPs ± standard deviation of three independent measurements in triplicates.

Particles	LL-III [g/L]	Equilibrium concentration [g/L]	Adsorption [g/g]
ION	0	0.001 ± 0.005	-0.001 ± 0.005
ION	0.25	0.135 ± 0.03	0.115 ± 0.034
ION	0.5	0.317 ± 0.065	0.183 ± 0.065
ION	1	0.755 ± 0.090	0.245 ± 0.090
ION	2	1.755 ± 0.080	0.245 ± 0.080
ION@PAA	0	-0.002 ± 0.006	0.002 ± 0.06
ION@PAA	0.25	0.007 ± 0.007	0.243 ± 0.007

ION@PAA	0.5	0.138 ± 0.038	0.362 ± 0.038
ION@PAA	1	0.448 ± 0.190	0.512 ± 0.190
ION@PAA	2	1.247 ± 0.221	0.753 ± 0.221
ION@P(AA-co-MAA)	0	-0.002 ± 0.002	0.002 ± 0.002
ION@P(AA-co-MAA)	0.25	0.056 ± 0.016	0.194 ± 0.016
ION@P(AA-co-MAA)	0.5	0.161 ± 0.042	0.339 ± 0.042
ION@P(AA-co-MAA)	1	0.509 ± 0.045	0.491 ± 0.045
ION@P(AA-co-MAA)	2	1.390 ± 0.064	0.610 ± 0.064
ION@PMAA	0	0.001 ± 0.008	-0.001 ± 0.008
ION@PMAA	0.25	0.049 ± 0.026	0.201 ± 0.026
ION@PMAA	0.5	0.255 ± 0.048	0.245 ± 0.048
ION@PMAA	1	0.630 ± 0.045	0.370 ± 0.045
ION@PMAA	2	1.585 ± 0.126	0.415 ± 0.126
ION@PAM	0	0.000 ± 0.005	0.000 ± 0.005
ION@PAM	0.25	0.150 ± 0.054	0.10 ± 0.054
ION@PAM	0.5	0.340 ± 0.013	0.160 ± 0.013
ION@PAM	1	0.857 ± 0.068	0.143 ± 0.068
ION@PAM	2	1.851 ± 0.052	0.149 ± 0.052

Table S2. Semi-quantitative ATR-FTIR analysis from Figure 1e, showing the integral (area under the curve, arbitrary units) for each particle system within the respective analyzed regions with the aliphatic-to-carboxyl integral ratio.

Particles	Integral aliphatic region (3000 – 2800 cm^{-1})	Integral carboxy region (1795 – 1345 cm^{-1})	Ratio (aliphatic/carboxy region)
ION@PAA	6.08	15.0	0.41

ION@P(AA-co-MAA)	7.50	13.6	0.55
ION@PMAA	5.92	8.02	0.74

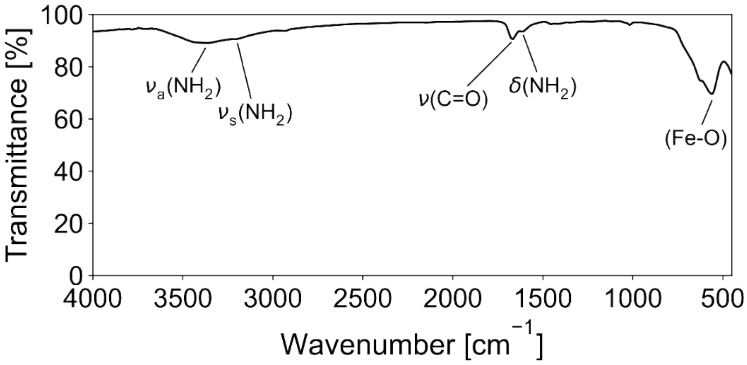

Figure S3. ATR-FTIR spectrum of ION@PAM showing the most characteristic peaks.

Table S3. Desorption of LL-III on polymer-coated and bare IONPs following three consecutive washing steps with PBS expressed as cumulative average desorption in percent of initially loaded LL-III ± standard deviation of three independent measurements in triplicates.

Particle	Wash	Cumulative average desorption [% of loaded LL-III]
IONPs	1	25.3 ± 17.0
IONPs	2	31.2 ± 8.29
IONPs	3	48.7 ± 14.1
ION@PAA	1	27.6 ± 13.5
ION@PAA	2	34.9 ± 11.5
ION@PAA	3	37.5 ± 9.86
ION@P(AA-co-MAA)	1	12.2 ± 7.80
ION@P(AA-co-MAA)	2	20.7 ± 7.65
ION@P(AA-co-MAA)	3	23.7 ± 9.14
ION@PMAA	1	16.5 ± 6.16
ION@PMAA	2	24.0 ± 12.7

ION@PMAA	3	27.8 ± 17.0
ION@PAM	1	26.9 ± 7.87
ION@PAM	2	35.9 ± 11.7
ION@PAM	3	38.2 ± 12.0

Table S4. Adsorption isotherm of LL-III on ION@P(AA-co-MAA), including the initial loading at various peptide concentrations and the desorption following the indicated washing step ± standard deviation of three independent measurements in triplicates.

LL-III [g/L]	Equilibrium concentration [g/L]	Adsorption [g/g]
0	-0.002 ± 0.003	0.002 ± 0.003
0.025	0.003 ± 0.002	0.023± 0.002
0.05	0.006 ± 0.004	0.044 ± 0.004
0.1	0.009 ± 0.014	0.091 ± 0.014
0.25	0.056 ± 0.017	0.194 ± 0.017
0.5	0.161 ± 0.047	0.339 ± 0.047
1	0.509 ± 0.049	0.492 ± 0.049
2	1.390 ± 0.070	0.610 ± 0.070
4	3.177 ± 0.093	0.823 ± 0.093
Wash 1		
0	-0.001 ± 0.001	0.001 ± 0.001
0.025	0.000 ± 0.002	0.022 ± 0.002
0.05	0.007 ± 0.004	0.043 ± 0.004
0.1	0.012 ± 0.013	0.089 ± 0.013
0.25	0.074 ± 0.027	0.176 ± 0.027
0.5	0.193 ± 0.041	0.307 ± 0.041
1	0.592 ± 0.058	0.408 ± 0.058

2	1.477 ± 0.037	0.523 ± 0.037
4	3.368 ± 0.042	0.632 ± 0.042
Wash 2		
0	-0.001 ± 0.001	0.001 ± 0.001
0.025	0.004 ± 0.003	0.021 ± 0.003
0.05	0.009 ± 0.005	0.041 ± 0.005
0.1	0.017 ± 0.010	0.083 ± 0.010
0.25	0.094 ± 0.026	0.156 ± 0.026
0.5	0.225 ± 0.034	0.275 ± 0.034
1	0.635 ± 0.052	0.365 ± 0.052
2	1.510 ± 0.039	0.490 ± 0.039
4	3.463 ± 0.052	0.537 ± 0.052
Wash 3		
0	0.001 ± 0.001	-0.001 ± 0.001
0.025	0.005 ± 0.003	0.020 ± 0.003
0.05	0.010 ± 0.005	0.040 ± 0.005
0.1	0.019 ± 0.012	0.081 ± 0.012
0.25	0.104 ± 0.030	0.146 ± 0.030
0.5	0.240 ± 0.029	0.260 ± 0.029
1	0.638 ± 0.055	0.362 ± 0.055
2	1.528 ± 0.018	0.472 ± 0.018
4	3.515 ± 0.038	0.485 ± 0.038

Table S5. Summary of adsorption kinetics of LL-III on ION@P(AA-co-MAA) over time. Samples were taken at the indicated timepoint. Adsorption is reported as average ± standard deviation of three independent measurements in triplicates.

Timepoint [h]	Adsorption [g/g]
0.008333333	0.19 ± 0.05
0.016666667	0.24 ± 0.01
0.033333333	0.23 ± 0.03
0.066666667	0.22 ± 0.05
0.1	0.24 ± 0.07
0.5	0.28± 0.06
1	0.32 ± 0.05
4	0.33 ± 0.04
24	0.32 ± 0.01

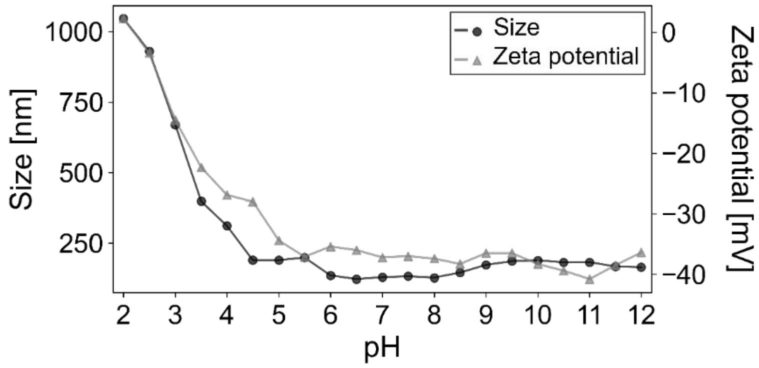

Figure S4. Hydrodynamic diameter and zeta potential of ION@(AA-co-MAA) as a function of pH obtained in ultrapure water adjusted to the indicated pH.

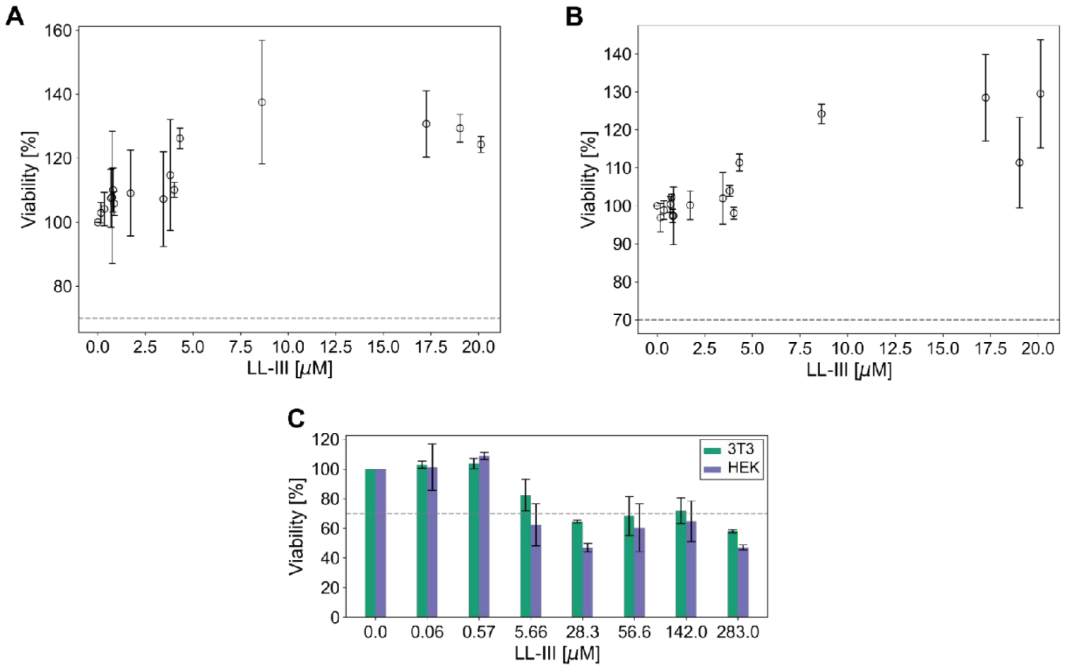

Figure S5. Cytotoxicity of LL-III-loaded ION@P(AA-co-MAA) on (A) 3T3 and (B) HEK cells as well as cytotoxicity of (C) only LL-III on 3T3 and HEK cells. Error bars represent the standard deviation of three independent measurements in triplicates.

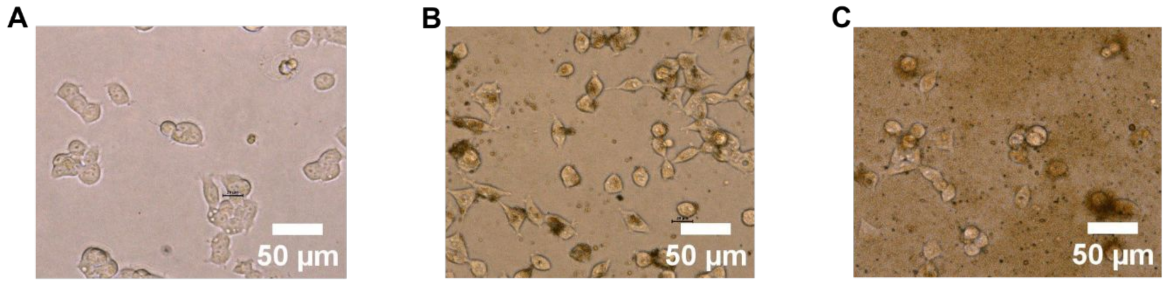

Figure S6. Micrographs (40 x magnification) obtained before analyzing cell viability. (A) Control HEK cells. (B) HEK cells treated with 50 μg/mL ION@P(AA-co-MAA). (C) HEK cells treated with 19 μM LL-III loaded on ION@P(AA-co-MAA).

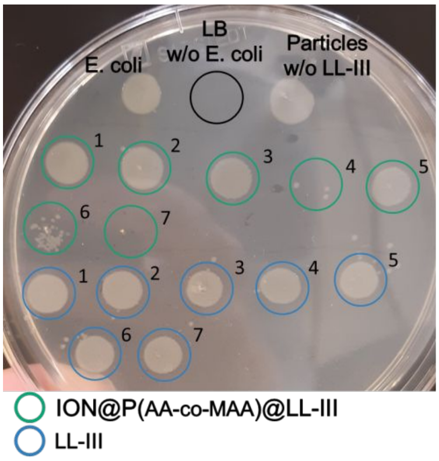

Figure S7. Bacterial growth on an LB agar plate using a drop assay evaluating the efficacy of LL-III-loaded ION@P(AA-co-MAA) (green circles) and free LL-III (blue circles) with different LL-III concentrations: 1 = 0.510 µM, 2 = 1.13 µM, 3 = 2.04 µM, 4 = 3.57 µM, 5 = 4.08 µM, 6 = 5.10 µM, 7 = 6.12 µM. Controls are indicated as *E. coli* (only *E. coli* in LB+Kanamycin medium), LB w/o *E. coli* (growth medium only), Particles w/o LL-III [ION@P(AA-co-MAA) without LL-III loaded].
